# Impact of hearing protection devices on sound localization performance

**DOI:** 10.3389/fnins.2014.00135

**Published:** 2014-06-11

**Authors:** Véronique Zimpfer, David Sarafian

**Affiliations:** ^1^French-German Research Institute of Saint-Louis (ISL), Acoustics and Protection of Soldier GroupSaint-Louis, France; ^2^Institut de Recherche Biomédicale des Armées, Département Action et Cognition en Situation OpérationnelleBrétigny sur Orge, France

**Keywords:** sound localization, HRTF, hearing protection device, spectral cues, behavioral method

## Abstract

Hearing Protection Devices (HPDs) can protect the ear against loud potentially damaging sounds while allowing lower-level sounds such as speech to be perceived. However, the impact of these devices on the ability to localize sound sources is not well known. To address this question, we propose two different methods: one behavioral and one dealing with acoustical measurements. For the behavioral method, sound localization performance was measured with, and without, HPDs on 20 listeners. Five HPDs, including both passive (non-linear attenuation) and three active (talk-through) systems were evaluated. The results showed a significant increase in localization errors, especially front-back and up-down confusions relative to the “naked ear” test condition for all of the systems tested, especially for the talk-through headphone system. For the acoustic measurement method, Head-Related Transfer Functions (HRTFs) were measured on an artificial head both without, and with the HPDs in place. The effects of the HPDs on the spectral cues for the localization of different sound sources in the horizontal plane were analyzed. Alterations of the Interaural Spectral Difference (ISD) cues were identified, which could explain the observed increase in front-back confusions caused by the talk-through headphone protectors.

## Introduction

Hearing protectors are traditionally divided into two categories: protectors in which the attenuation is constant and does not depend on the sound level, and protectors in which attenuation depends on the sound level. Only the latter allow users to communicate and to perceive sounds in the environment. This category can be further divided into two types:

- passive-protection systems, such as non-linear-attenuation earplugs. This type of protector is usually very effective for protection against impulse noise as the attenuation increases with the increasing peak pressure level of the sound. Non-linear-attenuation earplugs usually include a sound path with acoustic impedance depending on the particle velocity. For instance, it may consist of a cylindrical cavity perforated at either end, which is inserted into an earplug. The acoustic impedance of this cavity is related to its viscous resistance, which has a non-linear component proportional to the particle velocity (Dancer and Hamery, 1998);
- active protection systems such as electronic “talk-through” systems. In these systems, sound is recorded using an external microphone and played back at an appropriate level via a miniature loudspeaker placed inside the Hearing Protection Device (HPD) close to the listener's ear. The gain is reduced as the sound level increases.

Protectors in which the attenuation depends on the sound level protect the ear against loud, impulsive noise while allowing an almost unaltered perception of faint or moderate level sounds. These systems facilitate oral communication. However, their impact on the sound-localization performance is not well known. However, the ability to localize danger (warning sounds) may be vital and is therefore important, even when using HPDs.

In order to localize sound sources, listeners make use of various cues. These cues result from two physical phenomena, which occur as the sound propagates from its source to the listener's eardrum: reflections, which are added to the direct sound, and absorption. The resulting cues provide information concerning the distance of the source from the listener (Mershon and King, 1975; Zahorik et al., 2005). Moreover, acoustic effects introduced by the listener's body (including, in particular, the pinna, head, and torso) result in differences between the sounds received by the left ear and the right ear which are used to determine the angle of incidence of sounds (Blauert, 1983; Wightman and Kistler, 1992; Wightman, 1999; Cheng and Wakefield, 2001). In particular, interaural time differences (ITDs) and interaural level differences (ILDs) are used to localize sound sources to within a cone of confusion (Blauert, 1983; Hartmann, 1999; Carlile et al., 2005). However, ITDs and ILDs do not allow the listener to determine the elevation of the source. To perceive this elevation, listeners must make use of their implicit knowledge of the acoustic effects of their body on incoming sounds.

A previous study by Hofman et al. (1998) found that the insertion of a mold into the ear canal can have an impact on the listeners' ability to perceive the elevation of sound sources. Simpson et al. (2005) found modification in localization performance with linear HPDs in which the attenuation did not depend on the sound level. Lukas and Ahroon (2006) found degradation in localization performance with non-linear HPDs. To extend the findings of the previous studies (Lukas), we included active HPDs (talk-through system) in the present investigation. Sharon et al. (2007) showed a decreased performance in sound localization with a communication headset. (Gardner and Gardner, 1973) demonstrated that sound localization performance decreases with pinnae cavity occlusion. As described by Nicol (2010), many studies assess the sound localization performance in the horizontal plane which corresponds to the audiovisual horizon. But soldiers wearing HPDs move at all heights of the urban zone (for example, at the top of buildings) and need to localize sound also in the vertical plane. This is why we are interested in sound localization performance in azimuth and elevation. The goal of the present study was to investigate whether, and how, sound localization performance in azimuth and elevation is modified using active or passive hearing protection systems in which the attenuation depends on the sound level (e.g., Zimpfer et al., 2012). This sound localization performance was estimated using a psychophysical task method on different listeners. In the second part of the study, an analysis of the impact of the HPDs on the cues of the HRTFs was performed. This section highlights the distortion caused by the protection devices on the HRTFs.

The present study provides in particular some new contributions about localization performances in azimuth and elevation with level dependant HPD, and about a novel method using an artificial head to estimate localization performances with the same HPD.

## Behavioral experiment

### Materials and methods

In order to quantify the influence of hearing protectors on sound localization performance, we measured the latter with and without hearing protectors in a group of listeners.

#### Listeners

Twenty listeners (10 males, 10 females, aged 24–51, mean age = 33.5 ± 7 years) participated in the study. All the listeners had normal hearing, defined as age-compensated pure-tone hearing thresholds of less than 20 dB HL at octave frequencies between 250 and 8000 Hz (ANSI S3.6-2004). Listeners were also checked by otoscopy for abnormal cerumen build-up (corresponding to more than 1/8 of occlusion) inside the ear canal prior to the experiment. In compliance with the guidelines of the declaration of Helsinki and of the Huriet law regulating biomedical research on human subjects in France, the listeners provided written informed consent prior to their inclusion in the study. The listeners were paid (50€) for their participation.

#### Hearing protection device

**Figure 1:**
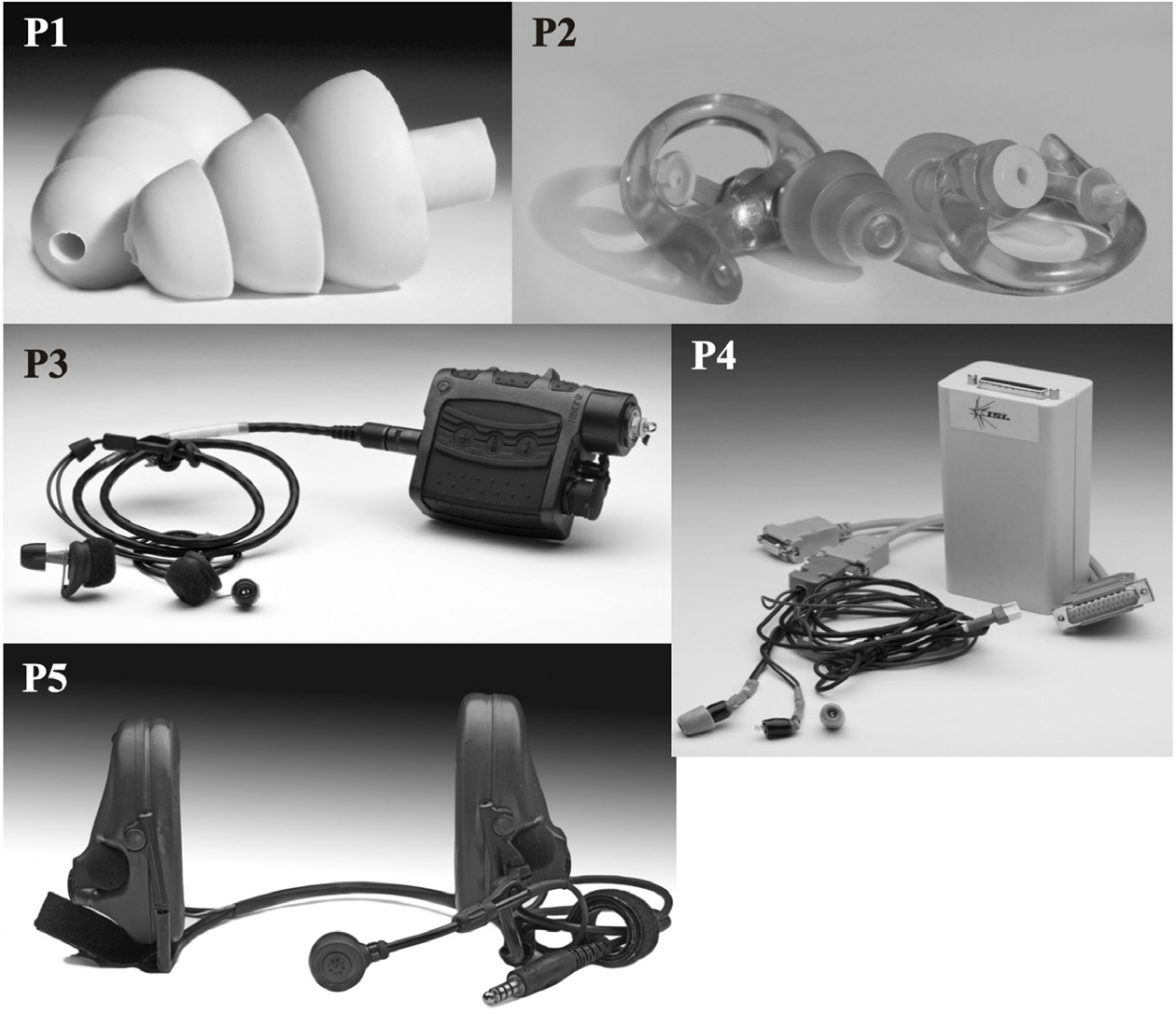
**Different HPDs tested in this study (see text for details)**.

Five HPDs (four earplugs and one earmuff)—two passive protectors (non-linear system) and three active protectors (talk-through system)—were tested (as shown in Figure 1):

- P1 is a commercial polymer earplug including an “ISL non-linear filter” with triple-flange design fit (3 sizes of earpieces).
- P2 is another polymer earplug including a Hocks-Noise-Braker® non-linear filter, with triple-flange design fit (3 sizes of earpieces).
- P3 is a commercial active earplug with a talk-through system and with modifiable foam tips (3 sizes).
- P4 is an ISL prototype earplug active talk-through system with modifiable foam tips (3 sizes).
- P5 is a commercial active earmuff with a talk-through system.

All the talk-through systems (earplug or earmuff) operate with two external microphones (one for each ear). For the three active systems, the tests were only carried out with the system in talk-through mode “ON,” which allowed a very moderate attenuation to be obtained in a quiet environment (under 70 dB of noise).

#### Apparatus

**Figure 2:**
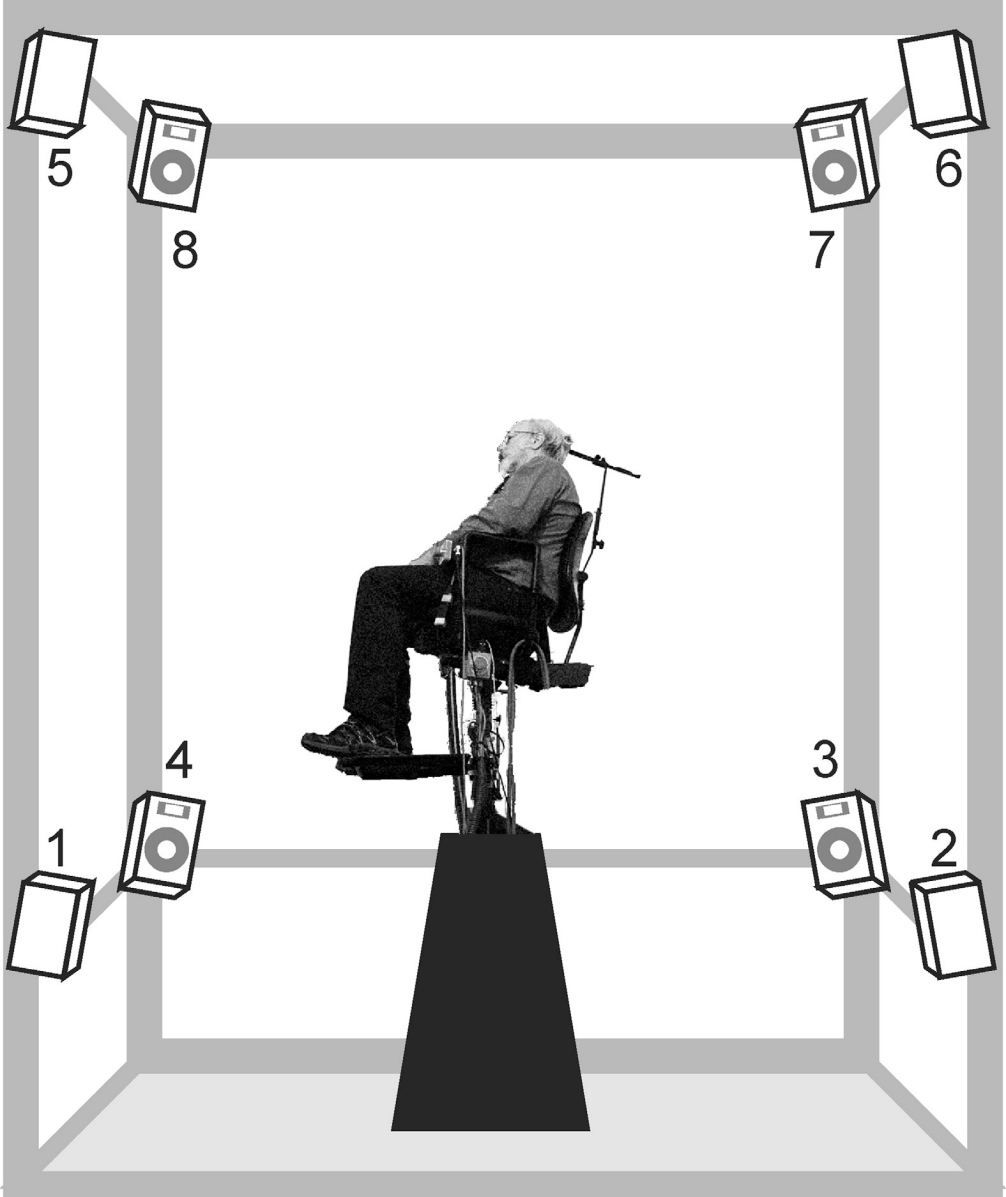
**Setup for sound-localization testing**.

In the center of a semi-anechoic chamber (polyhedron-shaped with a trapezoidal base (6 × 5.6 × 4.8 × 5 and 5.2 m high) with a carpet floor, eight loudspeakers were placed at the vertices of a cube having an edge dimension of 4 m. The background noise was measured with a Brüel and Kjaer type 4179 microphone and was in compliance with the ISO 4869-1: 1990 background sound level specifications. Listeners were individually seated on a chair placed in an elevated position (at an elevation of about 2 m, Figure 2) with the head placed in the center of this cube. They held a ball-shaped device with eight buttons on its surface, with each button corresponding to one speaker. The task of the listener was to press the button corresponding to the speaker which they identified as being the origin of the sound that was played to them. The number of correct responses and the test duration was recorded. This apparatus offers a 12.5% chance of having correct responses.

#### Auditory stimuli

On each trial, one of the eight loudspeakers emitted a brief signal; a 230 ms burst of wideband noise (see Butler and Planert, 1976). The acoustic stimuli were generated digitally at a 48 kHz sampling rate using a real-time processor (RX8; Tucker-Davis Technologies) with eight digital-to-analog converters (DACs). The output of each DAC was attenuated (PA5; Tucker-Davis Technologies) and routed to the corresponding loudspeaker via an amplifier (D-75A; Crown). The frequency response of each loudspeaker was equalized to provide the same acoustic signal at the listener's head location. The level of the signal (measured in the center of the cuboid speaker array) was set to 60 dB (SPL, lin.) for measurements without hearing protection and at 65 dB (SPL, lin.) for measurements with hearing protectors. In both cases, the stimulus was perfectly audible to all of the listeners. Indeed, with these noise levels, the different HPDs show no attenuation or only a very moderate one. To verify that the noise level was high enough, intelligibility tests using word lists were conducted with and without HPDs on each listener. These intelligibility tests were performed in the same anechoic chamber as the localization tests. The words were reproduced at the same level (65 dB) by one of the loudspeakers placed in front of the subject. During a test the subject had to recognize 15 words consisting of three phonemes. The rate of intelligibility was estimated by counting the number of correct phonemes (45 phonemes per test). The result of the intelligibility test was excellent with a rate of success of about 98% without and with HPDs. This proved that the sound level selected was sufficient for audibility.

#### Procedure

Prior to the experiment proper, listeners participated in three practice sessions, the goal of which was to acquaint them with the experimental apparatus and the task. During each practice session, eight sounds were presented sequentially to the listeners, each sound coming, in random order, from one of the eight loudspeakers. The listener's task was to identify the loudspeaker that emitted the sound. The purpose of these practice sessions was to reduce the training effect during the actual sessions.

During the actual experiment, the listeners participated in 13 test sessions. For three of these the listeners did not wear an HPDs; for the other 10 sessions, listeners wore HPDs (two test sessions for each HPD). The interest of these repeated sessions was to increase the reliability of the scores by averaging. During each of these sessions, 80 sounds (10 sounds per loudspeaker) were presented sequentially, in random order, to the listeners. The task was the same as during the practice sessions. To limit fatigue, sessions were separated by mandatory breaks of 10–15 min each, and listeners did not perform no more than four sessions per day. Four sessions with breaks lasted for about 50 min. Accordingly, the testing of each listener spanned 4 days. On the first day, otoscopic examination, and pure-tone audiometry tests were performed, after which the listener participated in three practice sessions and then in the first test sessions, without an HPD. Our intent was to begin and to finish with a session without HPDs in order to check the stability of the listener's localization performance. On the second day, each listener participated in four test sessions involving four different HPDs. On the third day, the listener performed three test sessions with different HPDs, and one test session without HPDs. On the fourth day, the listener performed three test sessions with different HPDs and finally, a session without HPDs. In order to avoid the effects of the order of testing of the different HPDs, a circular permutation of the listeners was arranged (see Table 1 for details). The entire experiment spanned 4 weeks.

**Table 1:** **Testing orders for days 2–4**.

	**Day 2**				**Day 3**				**Day 4**			
1	P1	P2	P3	P4	P5	N	P1	P2	P3	P4	P5	N
2	P2	P3	P4	P5	P1	P2	P3	N	P4	P5	P1	N
3	P3	P4	P5	P1	P2	P3	N	P4	P5	P1	P2	N
4	P4	P5	P1	P2	P3	P4	P5	N	P1	P2	P3	N
5	P5	P1	P2	P3	P4	P5	N	P1	P2	P3	P4	N

*The numbers (1–5) in the first column indicate different testing orders for the different HPDs (P1–P5). Each entry corresponds to one test session. Entries labeled “N” (for “None”) indicate test sessions during which listeners were tested with naked ears. The five testing orders of HPDS were a circular permutation of the listeners, so the testing order 1 was assigned to listeners 1, 6, 11, and 16 were assigned testing order 1, and so forth*.

In an attempt to provide the best possible fit for each listener, the size of the earpiece was selected on an individual basis, except for P5 (earmuff). Pictures of ears wearing the earpieces were taken in order to check the suitable insertion of each HPD throughout the tests. For the device labeled P3, the tightness of the fit was evaluated using an active (acoustic) system, which “beeped” every minute if the fit was not sufficiently tight. For four of the 20 listeners, a tight fit could not be obtained, regardless of which of the three available earpiece sizes was used. Therefore, these four listeners could not be tested with this device, and the mean results reported in paragraph 2.2 for P3 are based on the results from 16 listeners only (8 females and 8 males); for all the other HPDs, the mean results reported in the following section are based on 20 listeners.

### Results

For each hearing condition (N, P1–P5), we compared the two or three sessions which were realized. We observed the same mean number of correct responses for all listeners between the sessions with the same hearing condition. The differences between the sessions are not significant. For the following analyses, we represented the average between the sessions of same hearing condition.

**Figure 3:**
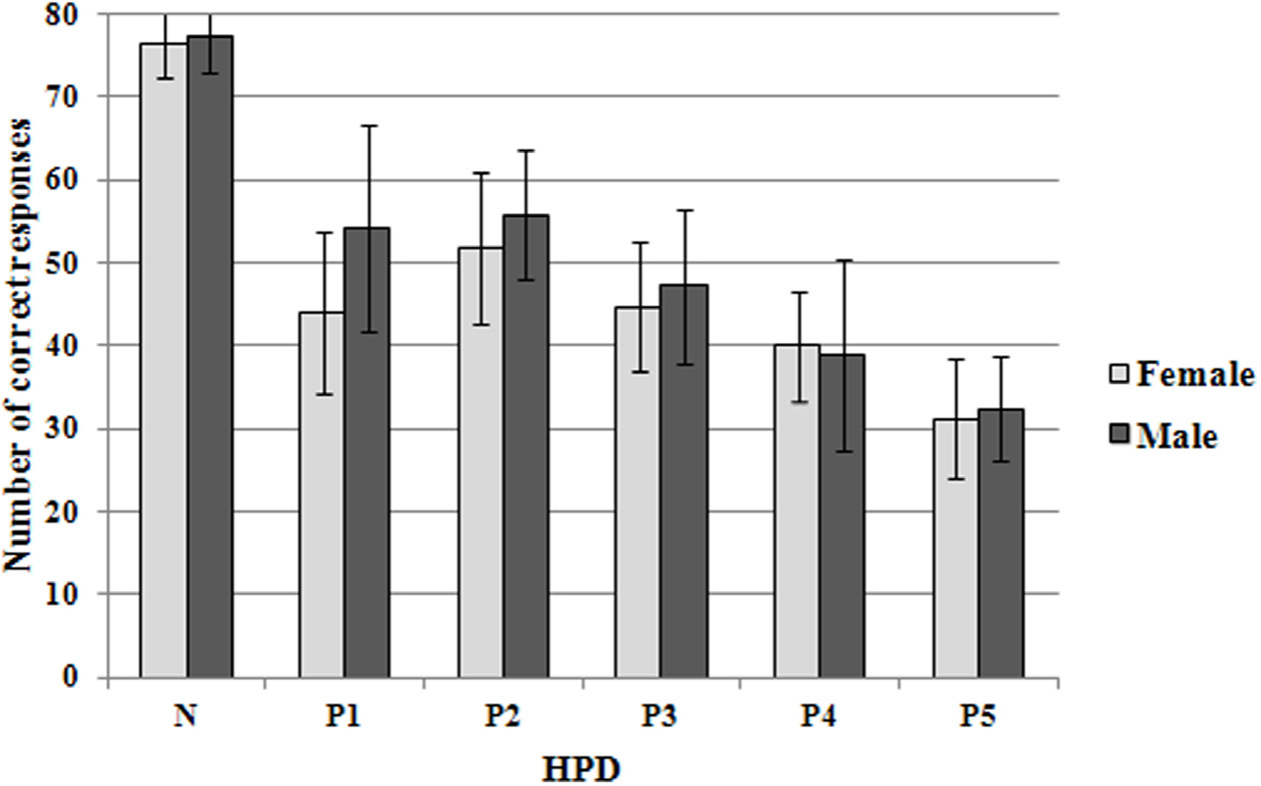
**Mean number of correct responses (for one session of 80 sounds) in the localization task for the different test conditions**. N: no HPD; P1–P5: for different HPDs. Error bars show ±1 standard deviation of the mean across listeners in the gender group considered (male or female).

Figure 3 shows the mean number of correct scores for each of the conditions tested in the localization task. The numbers of correct responses measured while the listeners were using HPDs (P1–P5) were always lower than those measured while the listeners were not wearing HPDs (N).

Without HPDs, the number of correct responses was analyzed using the analysis of variance (ANOVA) method with repeated measurements performed on one factor, i.e., the loudspeaker (eight positions). The results showed that the positions of the loudspeakers had no significant effect [*F*_(7, 152)_ = 1.3; *p* = 0.254]. The positions of the loudspeakers did not have a marked effect on sound localization performance.

The duration of each session (80 sounds) was recorded. Without the HPDs, the mean duration of a session was 215 s with a standard deviation of 17 s. On the contrary, with the HPDs this mean duration was 245 s with a standard deviation of 45 s. We noted an increase of the mean duration of a session as well as the standard deviation when the listener wears a hearing protection. An Analysis ANOVA showed a difference very significant (*p* < 0.001) between different hearing configuration (without or with HPDs). For the following analyses, the duration effect was not taken into account.

The mean individual number of correct responses, which was obtained by averaging the number of correct scores covering all the test conditions for each listener, ranged from 39 (/80) to 59 (/80). The standard deviations of these previous scores varied widely according to the different test conditions for each listener and ranged from 12 to 29. On the whole, no significant differences between the listeners were found [*F*_(19, 100)_ = 0.41, *p* = 0.98]. No main gender effect was detected (*p* > 0.3 for all hearing conditions), contrary to the lower performance of women in the spatial analysis of auditory scenes as described by Lewald and Hausmann (2013). Statistically, our scores did not depend on the listener effect.

The data (number of correct responses) were analyzed using a Two-Way repeated-measure analysis of variance (ANOVA). The results showed the significant main effect of the test condition factor (six levels: N, and P1 through P5; *p* < 0.001).

We chose to perform Two-Way ANOVA tests with software “R” only on the 16 listeners on whom the five HPDs were tested. Prior to this stage, the means of repetition were transformed by the function asin(sqrt(x)). The Mauchly sphericity test was significant with *p* = 0.040. So we applied the Greenhouse-Geisser correction which yielded a new value *F*_(2.9, 10.6)_ = 68.33 with *p* < 0.001. This correction did not change the significance of the first results. The test of effect size gave η^2^ = 0.85 which corresponds to a high effect with a f_cohen_ > 0.40. The multiple comparisons of means (Tukey Contrasts) test were performed. Table 2 gives the *p*-value of the planned pairwise comparisons. It shows significant differences between the sessions without HPDs and with all the HPDs. It shows no significant differences between P1, P2, and P3 and between P4 and P5. The lack of a statistically significant difference between conditions P1 and P2 may be related to the fact that these two protectors were of the same type (passive HPD). We can conclude from it that the active systems yielded lower scores (53 and 40% correct) than passive systems (63% correct). Besides, the active earmuff system yielded the lowest score (40% correct). The differences in average performance between the three types of HPDs (passive earplug, active earplug and active earmuff) were highly significant (*p* < 0.001), whatever the comparisons (passive earplug vs. active earplug, passive earplug vs. active earmuff and active earplug vs. active earmuff).

**Table 2:** **Results of pairwise comparisons covering the different test conditions, including the no-HPD (N) condition and each of the five HPD conditions (P1–P5) for 16 listeners**.

	***N***	**P1**	**P2**	**P3**	**P4**
P1	*p* < 0.001				
P2	*p* < 0.001	*p* = 0.306			
P3	*p* < 0.001	*p* = 0.990	*p* = 0.0799		
P4	*p* < 0.001	*p* = 0.019	*p* < 0.001	*p* = 0.108	
P5	*p* < 0.001	*p* < 0.001	*p* < 0.001	*p* < 0.001	*p* = 0.319

**Figure 4:**
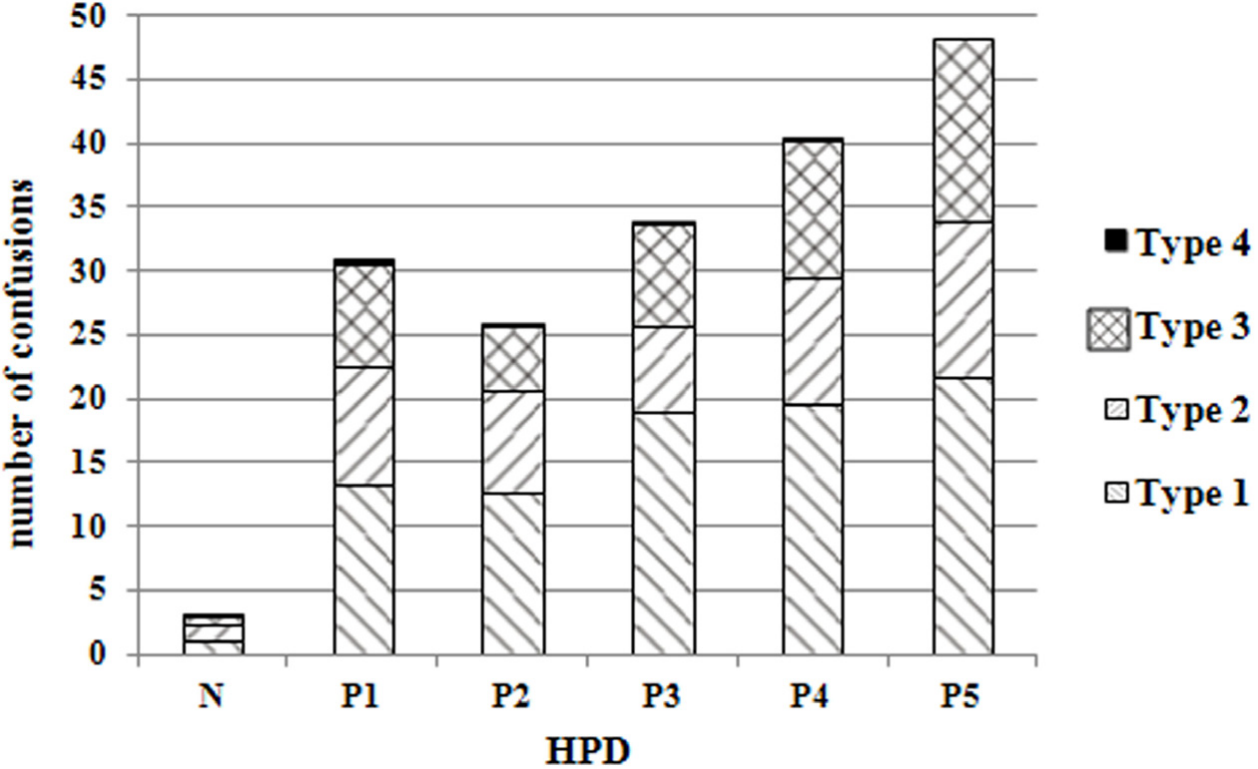
**Mean number of confusions (for one session of 80 sounds) for each test condition**. The different types of confusions are color-coded as follows. Type 1: up-down; Type 2: front-back; Type 3: combination of up-down and front-back; Type 4: combination of up-down, front-back, and left-right.

Figure 4 shows for one session (80 sounds) the mean number of different types of localization errors for each test condition. The most common types of errors were up-down confusions, followed by front-back confusions. These two types of confusions also occurred frequently in combination. Left-right confusions were rare and, when they did occur, they were almost always associated with up-down or front-back confusions. This is why they were grouped with the latter two types of confusions in this analysis. For these (left-right) confusions, the differences between the different conditions were not statistically significant (Table 3). For all the other types of confusions (i.e., front-back and up-down), highly significant differences were observed. For up-down confusions, pairwise comparisons between the different types of HPDs showed significant differences between all the test conditions, except for active earplugs vs. active earmuffs (Table 4); passive earplugs were found to produce fewer up-down confusions than active systems (earplugs or earmuffs). For front-back confusions, the planned pairwise comparisons showed significant differences between all the test conditions, except for passive earplugs vs. active earplugs (Table 5). The same remark can be made regarding front-back and up-down confusions (Table 6). No statistically significant difference could be found between passive earplugs and active earplugs, except for the elevation error. Whatever the confusion (up-down, front-back, and left-right) the difference between without HPD and with each HPD is significant.

**Table 3:** **Two-Way (test condition) repeated-measure analysis of variance (ANOVA) results for different types of confusion**.

**Confusion**	**ANOVA analysis**
Up-down	[*F*_(5, 110)_ = 20.36; *p* < 0.001]
Front-back	[*F*_(5, 110)_ = 13.65; *p* < 0.001]
Up-down + front-back	[*F*_(5, 110)_ = 19.4; *p* < 0.001]
Left-right	[*F*_(5, 110)_ = 0.97; *p* = 0.439]

**Table 4:** **Results of pairwise comparisons between the different test conditions for different types of HPDs for up-down confusions**.

	***N***	**Passive earplug**	**Active earplug**
Passive earplug	*p* < 0.001		
Active earplug	*p* < 0.001	*p* = 0.003	
Active earmuff	*p* < 0.001	*p* < 0.0001	*p* = 0.200

**Table 5:** **Results of pairwise comparisons between the different test conditions for different types of HPDs for front-back confusions**.

	***N***	**Passive earplug**	**Active earplug**
Passive earplug	*p* < 0.001		
Active earplug	*p* < 0.001	*p* = 0.0928	
Active earmuff	*p* < 0.001	*p* = 0.008	*p* < 0.001

**Table 6:** **Results of pairwise comparisons between the different test conditions for different types of HPDs for combined up-down and front-back confusions**.

	***N***	**Passive earplug**	**Active earplug**
Passive earplug	*p* < 0.001		
Active earplug	*p* < 0.001	*p* = 0.176	
Active earmuff	*p* < 0.001	*p* < 0.001	*p* < 0.001

### Discussion

The results of this study show that HPDs have a significant detrimental impact on sound localization performance. This was the case of all the systems tested in this study, including the passive earplugs, the active earplugs, and the active earmuff. The latter system caused the largest deterioration in sound-localization performance: the mean number of correct responses was 32 vs. the mean number of correct responses for the “naked ear” test condition which was 77. The percent-correct localization score obtained with this device (40%) was significantly lower than the scores achieved with any of the other devices tested in this study, including the other two active HPDs (earplugs). Passive earplugs were found to have the smallest impact on sound-localization performance, with an average score of 51 (/80), which still corresponds to a decrease of about 26 correct responses, compared to the “naked ear” condition. The scores for the two passive earplug systems tested here did not differ statistically. However, the score obtained with one of these two passive earplugs was also not significantly different from that measured with one of the two active earplugs. Another important observation was that HPDs increased both the front-back and up-down confusions. In particular, active systems distort the up-down localization perception. Front-back confusions are usually more detrimental than up-down confusions in real-life situations, as sounds of interest are usually located around, rather than above or below, the listener. Lastly, very few left-right confusions were observed and, when such confusions did occur, they were often accompanied by front-back or up-down confusions. These rare left-right confusions may be possibly due to a moment's inattention on the part of the listeners.

The detrimental effects of HPDs on sound-localization performance observed in this study can be explained by the fact that HPDs alter, or remove, cues used by listeners for localizing sounds, especially in the front-back and up-down dimensions. In particular, earplugs modify ear-canal resonances, which are known to introduce useful cues for sound localization in the form of spectral peaks and dips (Batteau, 1967; Hofman et al., 1998). Earmuffs alter spectral cues introduced by the pinna, which may explain why the earmuff-based protection system (P5) was found to be the most detrimental to sound-localization performance. Many localization confusions with active earmuff may be due to the fact that the pinna are hidden (Batteau, 1967; Hofman and van Opstal, 2003).

## Acoustic measurement

HRTFs provide a representation of the spectral modifications introduced by the listener's morphology (in particular, the torso, the head, and the pinna). These modifications can be determined by comparing the spectra of the recordings of a broadband noise (presented in the free field) at the entrance to the ear canal or close to the listener's eardrum, and the spectra of the recordings of the same signal obtained using a microphone placed at the location of the listener's head, in the listener's absence (Butler and Belendiuk, 1977; Blauert, 1983; Wightman and Kistler, 1989; Andéol et al., 2011).

### Materials and methods

To obtain information on the effects of the HPDs on the spectral cues for sound localization, we measured and compared the HRTFs using an artificial head in the horizontal plane without, and with, the HPDs in place. However, due to physical (volume and shape) constraints, the microphone used to measure the HRTFs could not be placed close to the listener's eardrum at the same time as an earplug. To solve the problem mentioned in Materials and methods, the HRTFs were measured using an artificial head built at ISL (Parmentier et al., 2000).

#### Hearing protection device

We used the same five HPDs as in the behavioral experiment.

#### Apparatus

The artificial head used is equipped with an IEC 711 compatible ear simulator (B&K 4157) in which the measured acoustic signal is close to that measured at a real eardrum. The outer ear and the ear canal are modeled using HeadAcoustics® materials. The artificial head was used to measure HRTFs without an HPD, and then with each of the HPDs. The measurements were performed in an audiometric cabin. Inside the cabin (2.6 × 4 × 2.2 m), the walls were covered with sound-absorbing material and the floor with a carpet.

#### Sound source

The sound source (loudspeaker) used for these measurements was located in the horizontal plane of the head. The distance between the loudspeaker and the artificial head was equal to 1.5 m. The 800 samples of HRTFs were recorded using the swept sine function with logarithmic steps [100 Hz–20 kHz]. The sound source level was fixed to 70 dB SPL, in order to prevent the active HPD from attenuating the sound as in the behavioral method.

#### Procedure

Measurements were performed in the horizontal plane for eight different orientations of the head (with respect to the sound source), spanning 360° in steps of 45°, and for each ear simultaneous (left and right). In the first orientation the head faces the source, which corresponds to the 0° angle. The sound source is fixed and the head is rotated to perform the measurements. For each orientation two measurements have been realized. After each measure, the HPD has been taken off and put back on the artificial ear. In order to avoid the parameter of the measurement chain, the reference measurement has been performed at the center of artificial head without the head. The HRTFs presented are the average of the two measurements. A comparison of the results of the acoustic measurement method with those of the previously mentioned psychophysical method cannot be strictly made. Indeed, the two methods do not analyze the same sound sources.

### Results

**Figure 5:**
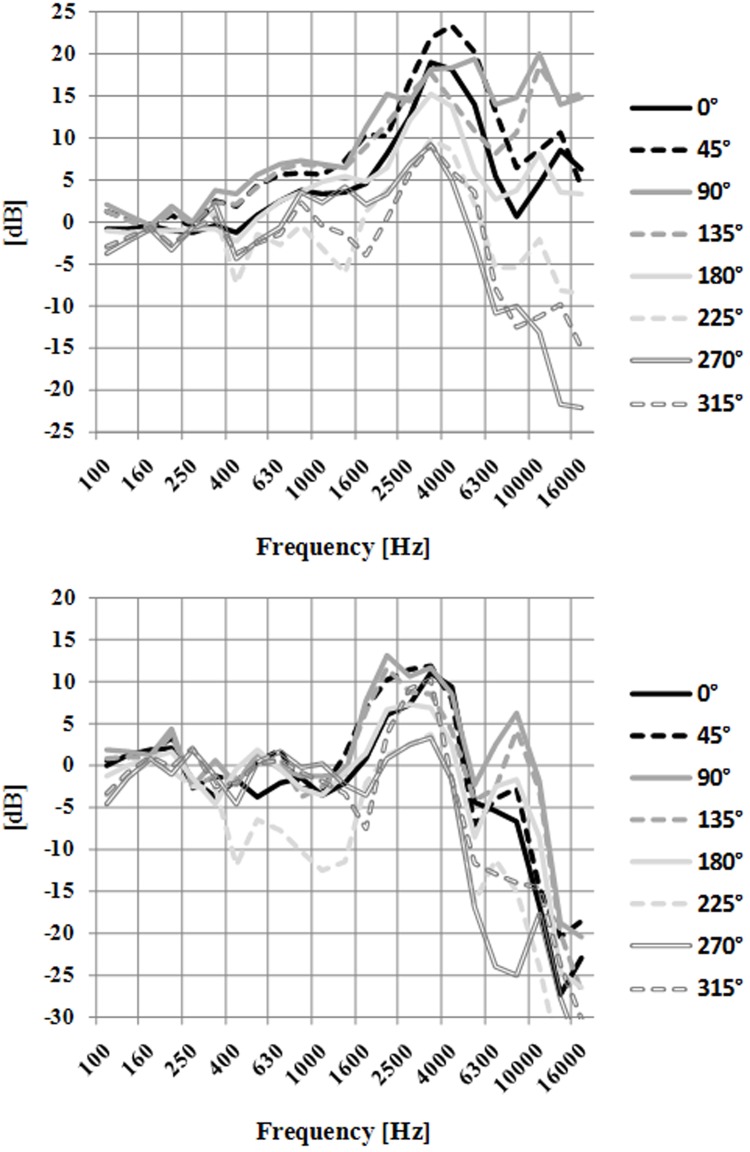
**HRTFs measured in the right ear, without an HPD in place at the top and with P5 in place at the bottom, for the eight orientations of the artificial head with respect to the noise source**. Different types of lines correspond to different orientations.

Figure 5 shows the HRTFs measured in the right ear, without an HPD and with the P5 device in place, for the eight orientations of the artificial head. It illustrates the effect of the head orientation on the HRTFs without the HPD in place. Similar figures were obtained on the left ear. In particular on the higher graph of Figure 5, it can be noted that as the orientation of the head with respect to the sound source varied from 0 to 315°, the sound power above 400 Hz initially increased, then decreased, thus reflecting the position of the right ear with respect to the source. Systematic variations in sound power as a function of the head orientation can also be observed at lower frequencies, down to about 400 Hz (Shaw, 1974). These orientation-dependent level variations in sound power levels at the eardrum correspond to the ILD which listeners potentially use to localize sound sources in the horizontal plane. The lower graph of Figure 5 shows that, with the P5 device in place, the HRTF in the 400 Hz–5 kHz frequency range varies only very little as a function of the head orientation (except for two orientations 225 and 270°). We can even note that for the 0 and 45° head orientations the HRTF curves are similar until 5 kHz. Eight curves of HRTF obtained with P5 are very different from those obtained without hearing protection (cf. Figure 5). This device also highlighted a small difference between the right and the left ears which may be due to the fact that this earmuff-based HPD was less symmetric than the others; in particular, as can be seen in Figure 1, this device featured a speech microphone only on the left side. The markedly reduced head-shadow effect produced by the earmuff of the HPD type suggests that listeners had to rely primarily on the ITD for left-right localization.

**Figure 6:**
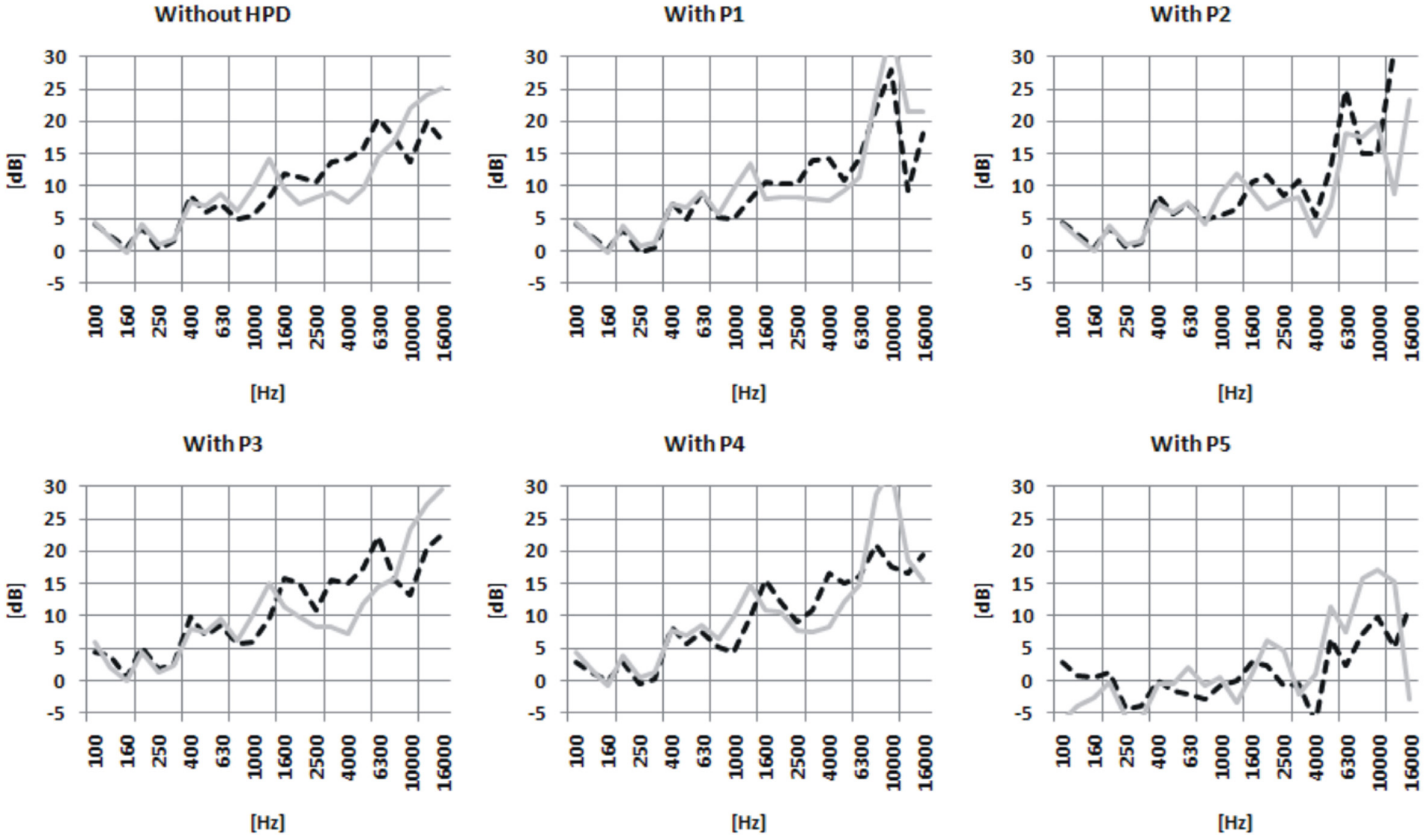
**The Interaural Spectral Difference (ISD) for head orientations of 45 (black, dashed line) and 135° (gray, solid line)**. The different panels correspond to different HPDs, or to the no-HPD condition (upper-left panel).

To obtain information about the relationship between the effects of HPDs on HRTFs and some possible front-back confusions, we were interested in HRTFs for the orientations of 45 and 135°. These two orientations correspond to front-right and back-right source locations, respectively. Specifically, we computed the Interaural Spectral Difference (ISD) which is the differences between the HRTFs measured in the left and right ears, for each of the two orientations. This was done for naked ears and for each HPD separately. The results, which are shown in Figure 6, illustrate the ISD cues that may have been available to the listeners for distinguishing between the front and back sources, for each HPD.

### Discussion about front-back confusion

**Figure 7:**
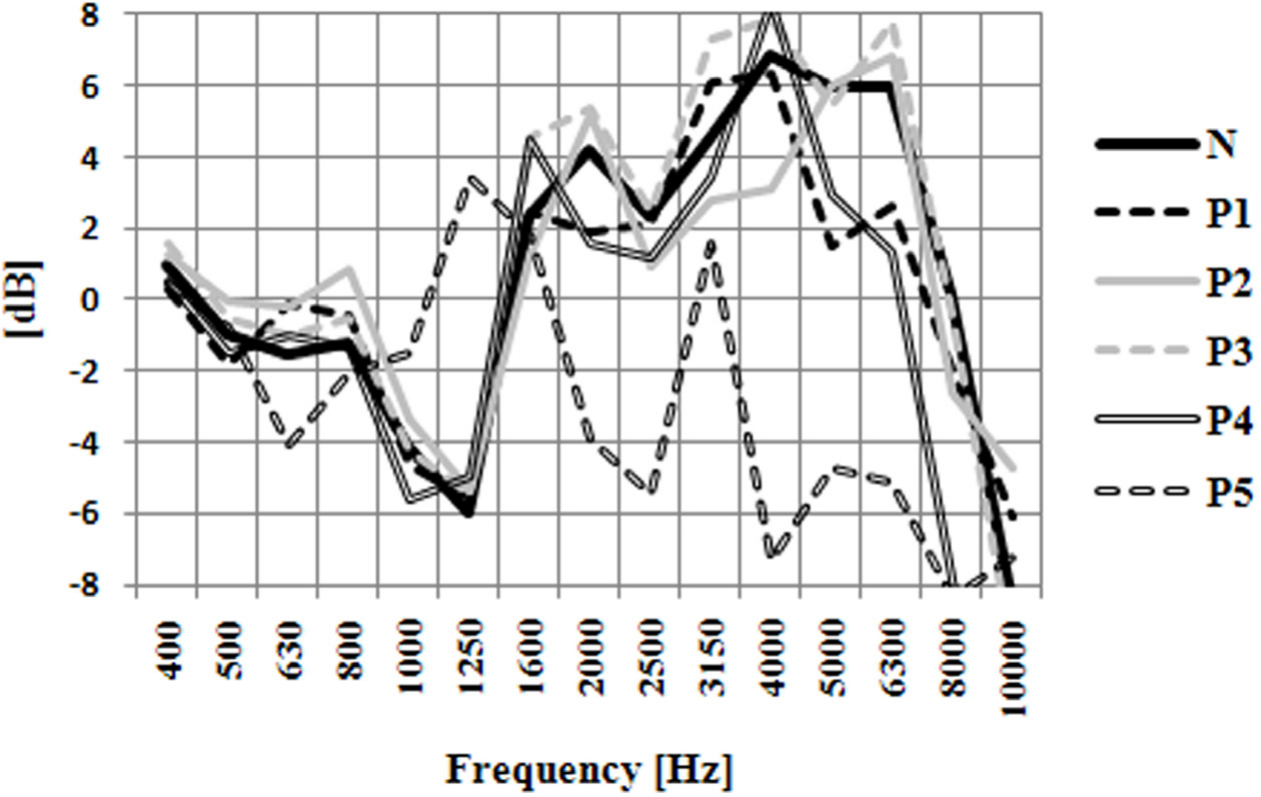
**Differences in the ISD cues between the 45 and 135° orientation for the right ear**.

It must be noted that, for the no-HPD condition (N), differences (up to 5 dB) between the ISD curves corresponding to the two orientations were observed over a wide range of frequencies (above approximately 500 Hz). Such differences provide a potential basis for the ability of listeners to distinguish between front and back locations. Differences between the two curves were also observed for the measurements performed with the HPDs in place. However, the magnitude and shape of these differences differed largely, depending on the type of HPD. This can be most easily seen in Figure 7, which shows the differences between the 45 and 135° ISD curves for the different HPDs, all superimposed on the same plot. It can be noticed that the ISD difference curves most similar to the reference (no-HPD) curve corresponded to P1, P2, and P3; for P4, and even more so for P5, large deviations from the reference curve were observed. This observation was confirmed quantitatively by comparing the mean of squared differences between the ISD difference curve for the naked ear and the ISD difference curves for each HPD, over the 0.5–10 kHz range (Table 7); the mean of squared difference was largest for P5. This indicates that the normal (naked-ear) pattern of the ISD cues for front-back distinctions was more severely altered by P5 than by the other HPDs. Table 7 shows the impact of HPDs on the HRTFs and the ISD cues.

**Table 7:** **Mean of squared difference between ISD-difference curves for the five HPDs (Figure 7)**.

	**P1**	**P2**	**P3**	**P4**	**P5**
Mean of squared difference (dB^2^)	4.05	3.81	1.74	12.24	57.90

As indicated in Table 7, the pattern of the ISD cues with respect to the distinction between the 45 and 135° orientations was the least altered by P3. Thus, the lowest proportion of front-back confusions was observed for P3, P2, and P1. For these three protectors, the differences between the ILD cues with and without hearing protection were the lowest. We could suppose that with these three HPDs the front-back confusions will be the lowest. Moreover, the information provided by Figure 7 and Table 7 also goes some way toward explaining the pattern of possible front-back confusions. We can note similarities between the two methods by comparing Figure 4 (behavioral method) with Table 7 (acoustic measurement method). Indeed, the three HPDs that are associated with the smallest mean of squared differences in Table 7, i.e., P1, P2, and P3 are the same three HPDs that were found to yield the smallest proportions of front-back confusions during the experiment. Besides, the worst result was obtained for the P5 protector for both methods (behavioral method and mean squared difference approach). These similarities between the two methods should be verified in future.

## Discussion and perspectives

The results of this study demonstrated the significant impact of the HPDs on sound-localization performance. The impact was more or less marked, depending on the type of HPD. It was less important for passive earplugs than for active systems. The decrease in sound-localization performance was the highest for the earmuff-based active system tested here. A larger number of localization errors, and especially, up-down confusions, were observed with active systems than with passive earplugs. However, front-back confusions were almost as numerous for passive earplugs (P1 and P2) as for one of the active earplug systems (P3). When comparing the physical dimensions of the different earplug devices with their results with respect to the localization performance, we note that the localization performance may possibly depend on the distance of the sound-pickup-point to the entrance of the ear canal.

Comparisons between the HRTFs measured with and without the HPDs provided some information about the origin of the decrease in localization performance in the horizontal plane due to HPDs. Specifically, by comparing the pattern of ILD cues used to distinguish between the 45 (front right) and 135° (back right) locations, we found that this pattern was more severely altered by P5 than by any of the other HPDs tested in this study. Moreover, this analysis showed that P1, P2, and P3 had a smaller impact on ISD cues than P4 and P5. These observations seem to correlate with the fact that localization performance was less degraded by P1, P2, and P3 than by P4 and P5. However, this correlation is, at the moment, more or less speculation, as it has to be confirmed by a new set of experiments conducted, with an identical setup for the measurement of HRTFs and the determination of the localization performance.

A limitation of the present study is due to the fact that HRTFs with HPDs (earplug) could not be measured in the human participant's ears. Ideally, HRTFs should have been measured while the participants were wearing the HPDs, for each HPD. Such measurements could not be performed due to the physical impossibility of fitting the HPD and the recording microphone into the ear canal. This is why HRTFs were measured using the artificial head. We are aware that this is not an ideal arrangement, and that future studies should try to resolve the technical difficulties associated with HRTF measurements in human participants wearing HPDs.

It is important to note that the HRTF measurements performed on an artificial head have shown spectral alterations caused by HPDs, which may explain the increase in front-back confusions observed for some HPDs. Once the measurement system is in place, HRTF measurements on an artificial head are less time-consuming than psychophysical tests which usually require multiple participants (in order to average out interindividual variability) and many stimulus presentations per participant. We have to demonstrate that the classification of the localization performance based on the HRTFs can be compared to the classification based on the psychophysical measurements. In this case, HRTF measurements using an artificial head may provide a fast(er) method for estimating the impact of HPDs on sound-localization performance. Specific alterations of the HRTF leading to particular errors in localization and measurement reproducibility could be interesting tracks for a next experiment. A limitation of this approach, however, is that it is based on a standard artificial head; it can only be used to predict average performance. HPDs may have a different impact on localization performance for different individuals, depending on morphological specificities (e.g., ear canal and/or pinna morphology) as well as on the quality of the fit. This poses an interesting challenge for future efforts to develop HRTF-based methods of predicting sound localization performance with HPDs.

### Conflict of interest statement

The authors declare that the research was conducted in the absence of any commercial or financial relationships that could be construed as a potential conflict of interest.
